# Sodium Glucose Cotransporter-2 Inhibitor Protects Against Diabetic Neuropathy and Nephropathy in Modestly Controlled Type 2 Diabetes: Follow-Up Study

**DOI:** 10.3389/fendo.2022.864332

**Published:** 2022-06-17

**Authors:** Fukashi Ishibashi, Aiko Kosaka, Mitra Tavakoli

**Affiliations:** ^1^Department of Internal Medicine, Ishibashi Clinic, Hiroshima, Japan; ^2^Exeter Centre of Excellence for Diabetes Research, National Institute for Health and Care Research (NIHR) Exeter Clinical Research Facility, and Institute of Biomedical and Clinical Sciences, University of Exeter Medical School, Exeter, United Kingdom

**Keywords:** SGLT2i, diabetic microvascular complication, protection against neuropathy and nephropathy, glycemic variability, extraglycemic factors, modest glycemic control, type 2 diabetes

## Abstract

**Aims:**

This three-year follow-up study aimed to elucidate whether sodium-glucose cotransporter-2 inhibitors (SGLT2is) have any protection against diabetic neuropathy and nephropathy in patients with type 2 diabetes *via* reducing variability in glycemia and extraglycemic factors or their averages.

**Methods:**

Two type 2 diabetic cohorts of 40 and 73 patients treated with or without SGLT2i along with 60 control subjects were recruited. Two diabetic cohorts matched for HbA1c levels and oral hypoglycemic agents other than SGLT2is underwent glycemic control with or without SGLT2is more than two years. The urinary albumin to creatinine ratio (ACR), estimated glomerular filtration rate (eGFR) every 3 months and neuropathy outcome measures and mean Z-score of 8 neurophysiological tests were determined at the baseline and endpoint. Glycemic variability, evaluated by the coefficient of variation of monthly measured HbA1c levels and casual postprandial plasma glucose (CPPG), and coefficient of variation and average of extraglycemic parameters in diabetic cohorts were determined.

**Results:**

The glycemic variability and variability of some extraglycemic factors in SGLT2i cohort were smaller than those in non-SGLT2i cohort. However, only smaller coefficient of variation of HbA1c improved some neuropathy outcome measures, and ameliorated eGFR decline. SGLT2i improved the Z-score of neurophysiological tests. The optimized changes in the blood pressure, HDL-cholesterol and uric acid by SGLT2i led to neurological and renal protection. SGLT2i decreased the prevalence of nephropathy significantly and the prevalence of neuropathy insignificantly.

**Conclusion:**

Over 3 years period, SGLT2i significantly improved some neuropathy outcome measures, mean Z-score of 8 neurophysiological tests, and attenuated nephropathy in modestly controlled type 2 diabetes by reducing glycemic variability and mean nonglycemic factors of diabetic microvascular complication.

## Introduction

Recent evidences have shown that sodium-glucose cotransporter-2 inhibitors (SGLT2is) exert a protection against diabetic nephropathy (1, 2) and cardiovascular disease (3) in addition to hypoglycemic effects. The trial using SGLT2i revealed renoprotection irrespective of the severity of nephropathy (4). To our knowledge, clinical trials so far did not address the effect of SGLT2is on diabetic neuropathy in type 2 diabetes. Although animal studies revealed amelioration of neuropathy (5), further clinical studies are needed for assessing the neuroprotection by SGLT2i in diabetic patients. The SGLT2is reduce glycemic variability mainly by suppressing postprandial hyperglycemia without hypoglycemia (6, 7). This may ameliorate the oxidative stress and chronic inflammation caused by high glycemic variability (8, 9). However, the influence of SGLT2i on the variability of extraglycemic factors and the benefit of their reduced CV and mean levels on diabetic neuropathy and nephropathy had never been studied. The current paper aimed to investigate the impact of SGLT2is on the neuropathy outcome measures (NOMs) along with nephropathy in patients with modestly controlled type 2 diabetes over three years period.

## Subjects and Methods

### Subjects

This was a longitudinal study. Among 1274 patients with type 2 diabetes who newly visited the Ishibashi Clinic between September 2010 and April 2019, we extracted patients whose HbA1c level at the first visit > 8.0%, and follow-up period longer than two years. Then, two diabetic cohorts undergoing glycemic control with or without SGLT2i along with other oral hypoglycemic agents (OHAs) were selected. The age, sex, and HbA1c levels during whole follow-up period between two cohorts with or without SGLT2i were matched. Sixty healthy control subjects with normal HbA1c levels (less than 5.9%) were enrolled and studied only at the baseline. Patients who were treated by insulin-sensitizing agents and sulfonylureas were given metformin and/or pioglitazone, and glimepiride or gliclazide, respectively. The exclusion criteria were following: any other clinically evident causes of neuropathy and nephropathy apart from diabetes, vitamin deficiency, corneal diseases, history of refractive surgery and use of hard contact lenses. Written informed consent was obtained from all subjects based on the Declaration of Helsinki. The ethics committee of the Ishibashi Clinic approved the protocol of the present research.

### Clinical and Laboratory Data

The body mass index (BMI), blood pressure, casual postprandial plasma glucose (CPPG), and HbA1c levels were measured monthly during the terms of study in patients with type 2 diabetes. In patients, the coefficient of variation (CV) of CPPG and HbA1c levels over the whole follow-up period was calculated to estimate glycemic variability. The serum lipid levels [LDL-cholesterol, HDL-cholesterol, and triglycerides], uric acid, high sensitivity C-reactive protein (hsCRP), estimated glomerular filtration rate (eGFR), urinary creatinine and albumin levels were assessed every 3 months in patients. CV of periodically measured extraglycemic factors was also calculated. An albumin-to-creatinine ratio (ACR) > 30 mg/g creatinine was labeled as nephropathy (10). In healthy control subjects all measurements were done only at the baseline.

### Assessment of Neuropathy and Neurophysiological Examinations

The severity of the neuropathy and neurological deficits was assessed using the modified neuropathy disability score (NDS) (11), which includes evaluating vibration, pinprick and temperature perception, and the ankle reflexes to establish the severity of neuropathy.

All patients with type 2 diabetes underwent neurophysiological examinations at the baseline and endpoint. Electrophysiology and nerve conduction velocity (NCV) studies were performed using an electromyography instrument (Neuropak S1, NIHON KOHDEN, Tokyo, Japan). The motor (MCV, median nerve) and sensory (SCV, sural nerve) NCV and their action potential amplitudes were determined.

The vibration perception threshold (VPT) was measured at the left medial malleolus using a biothesiometer (Biomedical Instruments, Newbury, OH, USA). The warm (WPT) and cold perception thresholds (CPT) at the dorsum of the foot were determined using a thermal stimulator (Intercross-200, Intercross Co., Tokyo, Japan). To assess the cardiovagal function of the autonomic nervous system, the CV of R-R intervals (CVR-R) was calculated from the R-R intervals of 200 samples on an electrocardiogram. The diabetic neuropathy was diagnosed based on the Toronto consensus of diabetic neuropathy (NDS > 2 and sural nerve SCV < 42 m/s) (12). We constructed mean Z-score of 8 neurophysiological tests (median MCV and amplitude, sural SCV and amplitude, VPT, WPT, CPT and CVR-R) for the comprehensive assessment of neuropathy; (individual value minus mean value of over-all study population) divided by SD. The sum of Z-scores of 8 neurophysiological tests was divided by 8.

### Corneal Confocal Microscopy

All subjects were examined using a Heidelberg Retina Tomograph III *in vivo* corneal confocal microscope with Rostock Corneal Module (Heidelberg Engineering, Heidelberg, Germany) (13). All patients underwent CCM at the baseline and endpoint. Six high-quality images of one eye per subject from Bowman’s layer were captured and analyzed to quantify the following corneal nerve fiber (CNF) morphological parameters: 1) CNF density, 2) CNF length, 3) corneal nerve branch density, and 4) beading frequency. All measurements were performed using ImageJ (Texelcraft, Tokyo, Japan). The examiners and image analysts were all blinded and masked to the study groups.

### Statistical Analyses

All statistical analyses were performed using the SPSS (version 26, Chicago, IL, USA), and p-value < 0.05 was considered statistically significant. A *post hoc* analysis of sample power using GPower 3.1 (http://gpower.software informer.com/3.1/) was conducted using a one-sided ANOVA (significance of 0.05) and the Kruskal-Wallis test for neuropathy and nephropathy measures. The subject’s statistical power ranged from 0.93 to 0.99. All values are presented as the mean ± standard error of the mean. All data sets were tested for the normality using the Shapiro-Wilk test. The differences between baseline and endpoint in two diabetic cohorts were assessed using the paired t test and Wilcoxon signed rank test for normally and non-normally distributed continuous variables, respectively, and χ^2^ test and McNemar test for normally and non-normally distributed categorical variables, respectively. Normally or non-normally distributed continuous variables at the baseline or endpoint among three cohorts were compared with one-way ANOVA and Kruskal-Wallis tests followed by the Mann-Whitney U test and Bonferroni correction, and χ^2^ test and McNemar test for normally and non-normally distributed categorical variables, respectively. The correlations between the changes in NOMs, ACR or eGFR during glycemic control, and variability or average of CPPG, HbA1c and extraglycemic factors in SGLT2i cohort were assessed using the multiple regression analysis. The trend in ACR and eGFR for three years was assessed by Jonckheere-Terpstra test. The benefits of SGLT2i and other OHAs, angiotensin receptor blocker (ARB) and statins for NOMs, ACR and eGFR were assessed using total type 2 diabetes population by multiple regression analysis.

## Results

### Demographic and Clinical Data

**Table 1** presents the demographic and clinical characteristics of study groups. The gender and age among the three cohorts were similar. At the endpoint, patients without SGLT2i were older than control subjects. Two diabetic cohorts were obese compared with control subjects, and the SGLT2i cohort had higher BMI than patients without SGLT2i (p < 0.001). The follow-up period of two diabetic cohorts was quite similar. Systolic (SBP) and diastolic blood pressure (DBP) at baseline in two diabetic cohorts and SBP at the endpoint in patients without SGLT2i were higher than control subjects. SBP and DBP in two diabetic cohorts were decreased during follow-up. SBP at the endpoint in SGLT2i cohort was lower than patients without SGLT2i. The average SBP and DBP in patients without SGLT2i were higher than control subjects. The average SBP in SGLT2i cohort was lower than cohort without SGLT2i. All values of CPPG and HbA1c levels were quite similar between two diabetic cohorts, and decreased same degree during follow-up period.

**Table 1:** Demographic data, clinical characteristics, and prescription of drugs at the baseline and endpoint in patients with type 2 diabetes treated with or without sodium glucose cotransporter-2 inhibitor and at baseline in control subjects.

	Patients with type 2 diabetes				Control subjects
	With SGLT2i		Without SGLT2i		
	Baseline	Endpoint	Baseline	Endpoint	Baseline
Number (Male/Female, %)	40 (70.0/30.0)	40 (70.0/30.0)	73 (67.1/32.9)	73 (67.1/32.9)	60 (66.7/33.3)
Age (year)	53.5±1.2	56.6±1.2*	53.7±0.88	56.8±0.86*,†	53.1±0.89
Body mass index (kg/m^2^)	30.1±0.72 ‡, §	29.8±0.76‡, §	24.8±0.43†	25.3±0.49‡,װ	22.9±0.53
Mean (CV%)		29.6±0.72‡,§ (1.7±0.1) ¶		24.9±0.46† (2.3±0.2)	
Follow up period (year)	–	3.10±0.12	–	3.16±0.11	–
Duration of diabetes (year)	12.7±1.1	15.8±1.1	10.7±0.9	13.8±1.0	–
Systolic blood pressure (mmHg)	139±1.5†	131±1.7*, #	144±1.9‡	137±1.4‡, *	132±1.8
Mean (CV%)		134±1.5 ¶ (6.3±0.26) ¶		138±0.97‡ (5.6±0.19)	
Diastolic blood pressure (mmHg)	83.1±1.0 ‡	78.2±1.3*	84.8±0.9‡	79.4±1.1*	77.8±0.87
Mean (CV%)		79.8±0.84 (7.5±0.32)¶		80.6±0.66† (6.6±0.22)	
CPPG (mg/dL)	192±11.8 ‡	162±9.1‡, *	199±8.2‡	168±6.9‡,װ	95.9±1.4
Mean (CV%)		158±6.4‡ (20.9±1.2) ¶		163±4.5‡ (25.8±1.1)	
HbA1c (%)	8.2±0.14‡	7.4±0.13‡,*	8.2±0.19‡	7.4±0.12‡,*	5.5±0.03
Mean (CV%)		7.6±0.12‡ (4.8±0.29) #		7.6±0.11‡ (7.1±0.47)	
LDL-cholesterol (mmol/L)	3.70±0.14**	3.72±0.14**, #	3.50±0.11	3.21±0.11††	3.23±0.09
Mean (CV%)		3.68±0.11†, # (12.0±1.1)		3.24±0.09 (13.1±0.69)	
HDL-cholesterol (mmol/L)	1.29±0.06‡, #	1.41±0.06‡,*	1.52±0.04**	1.49±0.05†	1.74±0.06
Mean (CV%)		1.37±0.06 ‡ (8.2±0.33) §		1.51±0.04**(10.0±0.29)	
Triglycerides (mmol/L)	2.27±0.34	2.04±0.20 **	2.22±0.20‡	2.11±0.21**	1.53±0.14
Mean (CV%)		2.00±0.16 ** (28.3±1.6)		2.00±0.15†(34.2±1.7)	
Uric Acid (μmol/L)	323±11.2	290±8.96*	296±9.6	314±8.3*	323±10.3
Mean (CV%)		296±8.9 (9.24±0.48)		308±8.1 (10.2±0.38)	
hsCRP (mg/dL)	0.140±0.026‡	0.131±0.022 ‡	0.086±0.011‡	0.092±0.016 ‡	0.0195±0.0022
Mean (CV%)		0.118±0.014 ‡ (46.3±5.1) #		0.091±0.013‡ (60.0±3.7)	
eGFR (mL/min)	74.2±2.18	74.0±2.28	80.3±2.27	73.1±1.96†*	81.2.±1.92
Mean (CV%)		74.8±2.21 (10.2±0.51)		74.9±1.89 (9.3±0.34)	
ACR (mg/gCr)	49.2±9.6‡	25.6±5.9‡,*	34.9±7.7‡	32.3±7.3‡	7.88±1.22
Mean (CV%)		37.1±7.4‡ (42.3±3.0)		28.5±6.1‡ (41.0±3.2)	
Hypoglycemic treatment					
None/SGLT2i/SU/ISA/α-GI/DPP4-I/ diet alone (%)	0/0/65/87.5/22.5/75/0	0/100*,§/65/87.5/22.5/75/0	2.7/0/63/79.5/19.2/74/1.4	0/0/63/80.8/20.5/76.7/0	**-**
ARB/statins (%)	52.5‡/12.5	52.5‡/22.5†	38.4‡/13.7**	57.5‡ */17.8†	1.7/1.7
SGLT2i no. (%)Dapagliflozin/Tofogliflozin/Ipragliflozin		27 (67.5) /10 (25.0)/3 (7.5)			

Data are the mean ± standard error of the mean in patients with type 2 diabetes with or without SGLT2i treatment at baseline and endpoint and control subjects at baseline.

^*^p < 0.001 compared with baseline, ^†^p < 0.01 compared with control subjects, ^‡^p < 0.001 compared with control subjects, ^§^p < 0.001 compared with patients without SGLT2i, ^װ^p < 0.01 compared with baseline, ^¶^p < 0.05 compared with patients without SGLT2i, ^#^p < 0.01 compared with patients without SGLT2i, ^**^P < 0.05 compared with control subjects, ^††^p < 0.05 compared with baseline.

ACR, albumin to creatinine ratio; α-GI, α-glucosidase inhibitor; ARB, angiotensin receptor blocker; CV, coefficient of variation; CPPG, casual postprandial plasma glucose; DPP4-I, dipeptidyl peptidase-4 inhibitor; eGFR, estimated glomerular filtration rate; HDL, high-density lipoprotein; hsCRP, high sensitivity C reactive protein; ISA, insulin-sensitizing agent (metformin or/and pioglitazone); LDL, low-density lipoprotein; SGLT2i, sodium-glucose cotransporter-2 inhibitor; SU, sulfonylurea (gliclazide or glimepiride).

All LDL-cholesterol values in SGLT2i cohort were higher than control subjects, and that at endpoint was higher than patients without SGLT2i. LDL-cholesterol in patients without SGLT2i was decreased during follow-up period. The mean LDL-cholestorol in SGLT2i cohort was higher than in patients without SGLT2i. The all HDL-cholesterol values in two diabetic cohorts were lower than control subjects, and that at baseline in SGLT2i cohort was lower than that in patients without SGLT2i. SGLT2i increased HDL-cholesterol significantly.

**Figure 1:**
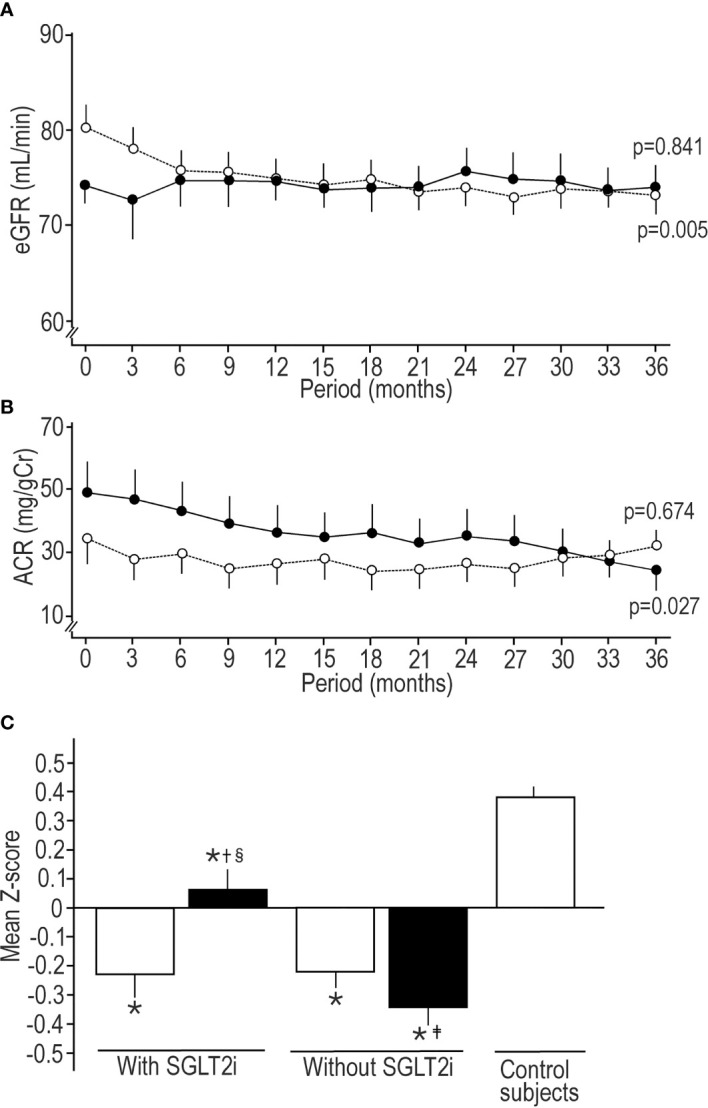
Sequential changes in estimated glomerular filtration rate **(A)** and albumin to creatinine ratio **(B)** in patients treated by SGLT2i (solid circle) and treated without SGLT2i (open circle). Values were mean ± standard error of the mean. The trend of decrease was assessed by Jonckheere-Terpstra test, and p value presents the significance of trend. **(C)** Comparison of mean Z-score of 8 neurophysiological tests (median motor nerve conduction velocity and amplitude, sural sensory nerve conduction velocity and amplitude, vibration perception threshold, coefficient of variation of R-R interval, warm perception threshold, and cold perception threshold) among patients treated with or without SGLT2i at the baseline and endpoint and healthy control subjects at the baseline. Open column; at the baseline, solid column; at the endpoint. Values were mean ± standard error of the mean. *p < 0.001 compared with control subjects, ^†^p < 0.001 compared with baseline, ^‡^p < 0.05 compared with baseline, ^§^p < 0.05 compared with patients without SGLT2i.

All triglycerides levels except for baseline in SGLT2i cohort in two diabetic cohorts were higher than control subjects. During follow-up period, uric acid in patients with SGLT2i was decreased, while increased in patients without SGLT2i. The all hsCRP levels in both diabetic cohorts were higher than control subjects. The treatment without SGLT2i decreased eGFR to the level less than control subjects, showing definite decreasing trend (**Figure 1A**), while in SGLT2i cohort eGFR did not deteriorate. All ACR values in the two diabetic cohorts were higher than control subjects. SGLT2i treatment showed significant decreasing trend in ACR (**Figure 1B**), but the treatment without SGLT2i did not. In SGLT2i cohort, the prescriptions of various OHAs other than SGLT2is were exactly the same between baseline and endpoint. Prescriptions other than SGLT2i at the endpoint were quite similar between two diabetic cohorts. The ARB was more prescribed at the baseline and endpoint in two diabetic cohorts than control subjects, and at the endpoint in patients without SGLT2i was more prescribed than at the baseline. The statins were more prescribed at baseline in patients without SGLT2i and at the endpoint in both diabetic cohorts than control subjects. Following SGLT2is were prescribed; dapagliflozin; 5mg or 10mg/day, tofogliflozin; 20mg/day and ipragliflozin; 25mg or 50mg/day (**Table 1**).

### CV of Periodically Measured Parameters

CV of BMI, CPPG, HbA1c, HDL-cholesterol, and hsCRP in patients treated by SGLT2i was significantly smaller than those in patients without SGLT2i, while CV of blood pressure in patients with SGLT2i was larger than those of patients without SGLT2i (**Table 1**).

### Comparison of CNF, Neurophysiological Tests and Nephropathy Between Baseline and Endpoint, or Among Three Cohorts

All CCM measures at the baseline and endpoint in both diabetic cohorts were less than control subjects. Beading frequency at the endpoint in patients without SGLT2i was fewer than at the baseline.

The NDS at baseline and endpoint in both diabetic cohorts was higher than control subjects, and modest glycemic control marginally increased NDS furthermore in both diabetic cohorts. The median nerve MCV at the baseline and endpoint in both diabetic cohorts was slower than that in control subjects. The median nerve amplitudes at the baseline in SGLT2i cohort and at the baseline and endpoint in patients without SGLT2i were lower than control subjects. SGLT2i significantly improved the median nerve amplitude to the higher level than that in patients without SGLT2i. In patients without SGLT2i the median nerve amplitude was significantly decreased during follow-up period. The glycemic control without SGLT2i reduced the sural nerve SCV, and at the endpoint it was lower than control subjects. The sural nerve amplitude in two diabetic cohorts was lower than control subjects all the time. Basal sural nerve amplitude in SGLT2i cohort was lower than patients without SGLT2i. The glycemic control by SGLT2i increased the sural nerve amplitude. The all VPT and CVR-R in two diabetic cohorts were altered compared with control subjects without changes during follow-up period. WPT at baseline in SGLT2i cohort and at the baseline and endpoint in patients without SGLT2i was compromized compared with control subjects. SGLT2i improved WPT at the endpoint, which was better than patients without SGLT2i. WPT deteriorated in patients without SGLT2i. CPT at the baseline and endpoint in patients without SGLT2i was inferior to control subjects (**Table 2**). Mean Z-scores of 8 neurophysiological tests at the baseline and endpoint in two diabetic cohorts were robustly lower than control subjects. SGLT2i treatment significantly increased mean Z-score, while it deteriorated in patients without SGLT2i (**Figure 1C**).

**Table 2:** Corneal nerve fiber measures, neurophysiological tests and microvascular complications at the baseline and endpoint in patients with type 2 diabetes treated with or without sodium glucose cotransporter-2 inhibitor and at the baseline in control subjects.

	Patients with type 2 diabetes				Control subjects
	With SGLT2i		Without SGLT2i		
	Baseline	Endpoint	Baseline	Endpoint	Baseline
**Corneal nerve fiber measures**					
Corneal nerve fiber density (no./mm^2^)	18.8 ± 0.57*	18.9 ± 0.50*	19.8 ± 0.61*	19.0 ± 0.63*	32.2 ± 0.74
Corneal nerve fiber length (mm/mm^2^)	10.6 ± 0.35*	10.6 ± 0.26*	10.5 ± 0.29*	10.3 ± 0.32*	15.4 ± 0.31
Corneal nerve branch density (no./mm^2^)	10.5 ± 0.46†	10.8 ± 0.44†	10.9 ± 0.55‡	11.1 ± 0.45†	13.4 ± 0.65
Beading frequency (no./mm)	19.2 ± 0.38*	18.3 ± 0.28*	19.8 ± 0.21*	19.1 ± 0.23*, §	23.1 ± 0.28
**Neurophysiological tests**					
Neuropathy disability score	4.70 ± 0.34*	4.95 ± 0.34*,װ	4.33 ± 0.26*	4.64 ± 0.28*	0.38 ± 0.08
MCV of median nerve (m/s)	52.8 ± 0.81*	53.0 ± 0.87*	52.7 ± 0.59*	52.9 ± 0.57*	58.1 ± 0.44
Amplitude of median nerve (mV)	6.00 ± 0.50‡	7.64 ± 0.60§,¶	6.37 ± 0.30‡	5.55 ± 0.30*, §	7.98 ± 0.31
SCV of sural nerve (m/s)	45.5 ± 0.84	46.3 ± 0.82	45.6 ± 0.56	44.7 ± 0.67 †, §	46.9 ± 0.53
Amplitude of sural nerve (μV)	7.73 ± 0.48*,#	9.66 ± 0.60 *,**	10.0 ± 0.56*	10.0 ± 0.71*	14.5 ± 0.84
VPT (μ/120 cycles/s)	3.60 ± 0.44*	3.33 ± 0.40 ‡	3.56 ± 0.20*	4.10 ± 0.28*	2.06 ± 0.24
CV of R-R interval (%)	3.36 ± 0.18†	3.08 ± 0.19*	3.08 ± 0.14*	2.98 ± 0.16*	3.90 ± 0.12
Warm perception threshold (W/m^2^)	-611 ± 36.7‡	-522 ± 32.0§,#	-610 ± 20.0‡	-640 ± 23.4*	-512 ± 11.8
Cold perception threshold (W/m^2^)	518 ± 21.2	493 ± 17.0	532 ± 12.2†	544 ± 15.2†	478 ± 11.1
Prevalence of nephropathy (%)	45.0#	22.5§	27.4	24.7	–
Prevalence of neuropathy (%)	22.5	17.5	20.5	28.8	–

Data are the mean ± standard error of the mean in patients with type 2 diabetes with or without SGLT2i treatment at the baseline and endpoint and control subjects at the baseline.

^*^p < 0.001 compared with control subjects, ^†^p < 0.05 compared with control subjects, ^‡^p < 0.01 compared with control subjects, ^§^p < 0.01 compared with baseline, ^װ^p <

0.05 compared with baseline, ^¶^p < 0.01 compared with patients without SGLT2i, ^#^p < 0.05 compared with patients without SGLT2i, ^**^P < 0.001 compared with baseline, ^††^p < 0.001 compared with patients without SGLT2i.

ACR, albumin to creatinine ratio; CV, coefficient of variation; MCV, motor nerve conduction velocity; SCV, sensory nerve conduction velocity; SGLT2i, odium-glucose cotransporter-2 inhibitor; VPT, vibration perception threshold.

The prevalence of nephropathy at baseline in patients treated by SGLT2i was higher than patients without SGLT2i, and SGLT2i significantly decreased the prevalence of nephropathy.The prevalence of neuropathy in both diabetic cohorts was not changed significantly under modest glycemic control, but insignificantly decreased in SGLT2i cohort and increased in patients without SGLT2i (**Table 2**).

**Table 3** shows the relationship between the improvement of NOMs or renal outcomes and the mean or CV of glycemic and extraglycemic factors in SGLT2i cohort after correction by sex, age and the duration of diabetes. WPT was improved related with high mean HDL-cholesterol, while high mean uric acid and CV of ACR negatively influenced on WPT. The median nerve amplitude deteriorated by high mean SBP and CV of HbA1c. The sural nerve amplitude was deteriorated by high mean SBP and CV of HbA1c. The high mean ACR led to a decrease in ACR during follow-up period. The eGFR change during SGLT2i treatment was influenced negatively by high mean SBP, DBP and CV of HbA1c. The high mean hsCRP negatively influenced median nerve amplitude and eGFR, but it did not reach the statistical significance (**Table 3**).

**Table 3:** Correlations between the changes in warm perception threshold, amplitude of median and sural nerve, albumin to creatinine ratio or estimated glomerular filtration rate and mean or coefficient of variation of glycemic and extraglycemic factors in patients with type 2 diabetes treated by sodium-glucose cotransporter-2 inhibitors.

Changes during the follow-up period (value at the endpoint – value at the baseline)										
	Warm perception threshold		Amplitude of median nerve		Amplitude of sural nerve		Albumin to creatinine ratio		Estimated glomerular filtration rate	
	Standard β	p	Standard β	p	Standard β	p	Standard β	p	Standard β	p
Sex	-0.054	0.738	-0.103	0.516	0.090	0.565	0.183	0.265	-0.144	0.379
Age	-0.183	0.279	-0.199	0.227	-0.237	0.148	-0.101	0.550	-0.078	0.642
Duration of diabetes	-0.129	0.444	-0.205	0.213	-0.208	0.203	-0.095	0.571	-0.159	0.347
**Means of parameters**										
Body mass index	-0.160	0.347	-0.288	0.077	-0.284	0.078	0.110	0.518	-0.159	0.352
Systolic blood pressure	0.094	0.638	-0.385	**0.042**	-0.439	**0.017**	0.053	0.791	-0.496	**0.010**
Diastolic blood pressure	-0.124	0.479	-0.253	0.133	-0.026	0.879	-0.147	0.400	-0.483	**0.004**
CPPG	0.244	0.218	-0.267	0.129	-0.214	0.221	-0.093	0.613	-0.062	0.736
HbA1c	0.135	0.475	-0.161	0.381	-0.247	0.171	-0.060	0.753	-0.166	0.383
LDL-cholesterol	0.210	0.199	-0.088	0.585	-0.138	0.382	0.042	0.800	-0.075	0.651
Triglycerides	-0.303	0.067	-0.096	0.560	0.254	0.113	-0.248	0.138	0.039	0.818
High sensitivity C-reactive protein	-0.007	0.966	-0.316	0.053	-0.084	0.612	-0.082	0.634	-0.327	0.052
eGFR	-0.283	0.191	0.048	0.820	0.166	0.428	0.125	0.568	0.077	0.725
Albumin to creatinine ratio	-0.039	0.823	-0.302	0.066	0.097	0.558	-0.533	**0.001**	0.020	0.909
Uric acid	-0.483	**0.007**	0.033	0.858	-0.188	0.298	-0.235	0.210	-0.193	0.305
**CV of parameters**										
Body mass index	0.200	0.266	0.252	0.148	0.218	0.206	0.198	0.272	0.124	0.496
Systolic blood pressure	-0.045	0.787	-0.037	0.817	-0.106	0.506	0.270	0.098	-0.228	0.165
Diastolic blood pressure	-0.009	0.956	0.178	0.283	-0.068	0.681	0.196	0.252	0.200	0.241
CPPG	0.089	0.599	0.018	0.915	0.005	0.976	-0.092	0.587	-0.269	0.106
HbA1c	0.069	0.692	-0.341	**0.038**	-0.419	**0.009**	**-**0.265	0.123	-0.493	**0.003**
LDL-cholesterol	-0.076	0.668	0.119	0.490	0.062	0.716	-0.106	0.550	-0.148	0.403
HDL-cholesterol	-0.085	0.606	-0.008	0.961	0.082	0.607	0.046	0.779	-0.249	0.127
Triglycerides	-0.048	0.770	0.122	0.448	0.164	0.300	0.109	0.510	0.075	0.653
High sensitivity C reactive protein	0.081	0.647	0.030	0.860	0.125	0.461	-0.071	0.689	0.057	0.749
eGFR	-0.116	0.596	-0.123	0.564	0.303	0.144	-0.094	0.668	-0.212	0.332
Albumin to creatinine ratio	-0.335	**0.039**	-0.022	0.895	0.124	0.437	-0.199	0.2430	-0.215	0.193
Uric acid	0.185	0.265	-0.024	0.885	0.096	0.550	-0.165	0.321	-0.014	0.936

Statistically significant correlations appear in boldface type. Change = value at the endpoint – value at the baseline. Correlations were corrected by clinical covariates (sex, age and duration of diabetes).

CPPG, casual postprandial plasma glucose; CV, coefficient of variation; eGFR, estimated glomerular filtration rate; HDL, high-density lipoprotein; LDL, low-density lipoprotein.

In total patients with type 2 diabetes the use of SGLT2i was beneficial for the improvement of WPT, median and sural nerve amplitudes, ACR and eGFR decline. Sulfonylurea deteriorated eGFR. The use of metformin, pioglitazone and α-glucosidase inhibitor was beneficial to sural and median nerve amplitude and eGFR, respectively. ARB seemed to decrease ACR and statins were beneficial to WPT (**Table 4**).

**Table 4:** Correlations between the changes in warm perception threshold, amplitude of median and sural nerve, albumin to creatinine ratio or estimated glomerular filtration rate and the use of various oral hypoglycemic agents, angiotensin receptor blockers or statins at the endpoint or clinical covariates in total type 2 diabetic patients treated with or without sodium-glucose cotransporter-2 inhibitors.

Changes (value at the endpoint – value at the baseline)										
	Warm perceptionthreshold		Amplitude ofmedian nerve		Amplitude ofsural nerve		Albumin tocreatinine ratio		Estimated glomerularfiltration rate	
	Standard β	p	Standard β	p	Standard β	p	Standard β	p	Standard β	p
Sex	0.023	0.812	-0.078	0.422	0.028	0.776	-0.046	0.630	-0.100	0.297
Age	-0.030	0.757	-0.046	0.636	0.072	0.457	0.096	0.320	0.031	0.750
Duration of diabetes	-0.120	0.213	0.011	0.908	-0.094	0.332	-0.059	0.539	-0.109	0.254
Mean body mass index	0.087	0.371	0.086	0.381	0.008	0.934	-0.101	0.300	0.165	0.090
**Oral hypoglycemic agents**										
SGLT2 inhibitor	0.412	**<0.001**	0.475	**<0.001**	0.364	**0.001**	-0.239	**0.028**	0.282	**0.009**
Sulfonylurea	-0.011	0.915	0.016	0.876	-0.163	0.110	-0.021	0.835	-0.234	**0.019**
Metformin	0.063	0.531	0.028	0.782	0.286	**0.004**	-0.051	0.617	0.109	0.275
Pioglitazone	0.037	0.718	0.394	**<0.001**	0.115	0.268	0.091	0.378	-0.068	0.503
DPP-4 inhibitor	-0.133	0.173	-0.128	0.192	-0.042	0.670	-0.166	0.087	0.053	0.582
α-glucosidase inhibitor	0.038	0.692	-0.115	0.235	-0.012	0.903	-0.089	0.354	0.207	**0.029**
**Angiotensin receptor blockers**	-0.032	0.742	0.039	0.692	-0.080	0.416	-0.208	**0.031**	0.040	0.680
**Statins**	0.235	**0.020**	0.075	0.464	0.170	0.096	0.022	0.827	0.036	0.724

Statistically significant correlations appear in boldface type. Correlations were corrected by clinical covariates (sex, age, duration of diabetes and mean body mass index).

DPP-4, dipeptidyl peptidase-4; SGLT2, sodium-glucose cotransporter-2.

## Discussion

Although the strict glycemic control is an important strategy for the primary and secondary prevention of diabetic neuropathy and nephropathy (14, 15), growing attention has been paid to the potential role of glycemic variability in developing neuropathy and nephropathy. Along with hyperglycemia and glycemic variability, risk factors for neuropathy and nephropathy include body weight, blood pressure, lipid levels (16), and uric acid (17). Although the variability of these extraglycemic factors influenced on renal outcomes in type 2 diabetes (18), their role in developing neuropathy had not been fully investigated. The current hypoglycemic strategies are not optimal and are associated with weight gain and hypoglycemia, resulting in increased glycemic variability. Glycemic variability has emerged as another measure of glycemic control, which might constitute more reliable predictor of diabetic complications than mean HbA1c levels (19). Until a few years ago, SGLT2i had been mostly used in obese type 2 diabetes patients as second or later line OHA. Based on lowering postprandial hyperglycemia without hypoglycemia, SGLT2i would be expected to reduce glycemic variability and have advantages over other OHAs (20, 21). Besides hypoglycemic effect, SGLT2i reduces body weight, blood pressure, uric acid and triglycerides, and may increase LDL-cholesterol and HDL-cholesterol (22). However, the benefit of SGLT2i for reducing the variability of extraglycemic factors had not been investigated.

The short term studies confirmed the reduction of glycemic variability by SGLT2i in type 1 (7) and type 2 diabetes (23) using continuous glucose monitoring (CGM). However, for elucidating the benefit of SGLT2is for neuropathy and nephropathy by reducing glycemic variability, the follow-up period over a couple of years is necessary. The glycemic variability calculated from monthly measured HbA1c and CPPG levels in the present study was more representative than CGM parameters evaluated merely at the endpoint. For assessing the benefit of SGLT2i in reducing glycemic variability and CV of extraglycemic factors for diabetic neuropathy and nephropathy, the present study recruited two diabetic cohorts treated with or without SGLT2is, who maintained quite similar HbA1c levels, and were prescribed similar OHAs other than SGLT2i during follow-up period. So far there had been no follow-up study recruiting these two cohorts with type 2 diabetes for clarifying the benefit of reducing CV and mean of glycemia and extraglycemic factors against neuropathy and nephropathy by SGLT2i. In type 1 diabetes the long-term HbA1c variability was linked to neuropathy independent of mean HbA1c (24). We reported that glycemic variability assessed by CV of long term HbA1c levels and CPPG compromized NOMs in type 2 diabetes (25).

Ipragliflozin improved the sciatic nerve MCV in diabetic Torii fatty rat (5) and prevented hypersensitivity and intraepidermal nerve fiber loss in the streptozotocin-induced diabetic rats (26). However, no trials assessed the influence of SGLT2is on diabetic neuropathy. Thus, clinical data are needed for establishing the neuroprotection by SGLT2i independent of glycemic levels. In our study the baseline neurophysiological tests except for sural nerve amplitude and CNF parameters in two diabetic cohorts under qual glycemic control with or without SGLT2i treatment were similarly compromized. In patients treated without SGLT2i there was no improvement in these measures, and some tests deteriorated, resulting in decreased mean Z-score of neurophysiological tests. In contrast, in SGLT2i cohort no deterioration of NOMs was found, and some NOMs and Z-score of neurophysiological tests were significantly improved. This means that under modest glycemic control (mean HbA1c; 7.6%) and obesity-induced chronic inflammatory environment, SGLT2i was neuroprotective in type 2 diabetes. For WPT high mean HDL-cholesterol was beneficial, and high mean uric acid was harmful. The increase in HDL-cholesterol and decrease in uric acid by SGLT2i may improve WPT. The improvement of median nerve amplitude was inversely related with mean SBP and CV of HbA1c. The sural nerve amplitude was improved negatively related with mean SBP and CV of HbA1c. The decrease in SBP and CV of HbA1c by SGLT2i might result in the neuroprotection. However, SGLT2i did not significantly decrease the prevalence of neuropathy when labeled on the Toronto consensus of diabetic neuropathy (NDS > 2 and sural nerve SCV < 42 m/s), because SGLT2i did not improve NDS nor sural nerve SCV.

The previous studies using selective SGLT2is consistently revealed to attenuate diabetic nephropathy (1, 2) in type 2 diabetes. In the present study SGLT2i robustly decreased ACR, but treatment without SGLT2i did not. In patients without SGLT2i the eGFR was decreased during follow-up period, while in SGLT2i cohort eGFR did not deteriorate. There was clear decreasing trend in eGFR in patients without SGLT2i, and in ACR in patients with SGLT2i during follow-up period. These results indicated that SGLT2i exerted the renal protection in type 2 diabetes independent of HbA1c levels. The effect of SGLT2i on ACR tended to be greater with higher mean ACR as previously reported (27). The high mean SBP and DBP, and CV of HbA1c were related with eGFR decline. The significantly lower mean SBP and CV of HbA1c in SGLT2i cohort than patients without SGLT2i may prevent eGFR decline. The increased blood pressure variability was reported to predict eGFR decline and increase in albuminuria (28). However, the present study could not clarify the significant influence of blood pressure variability on renal outcomes, although blood pressure variability in SLLT2i cohort is slightly larger than that in cohort without SGLT2i.

The variability of HDL-cholesterol and triglycerides (28) has been reported as a risk factor for diabetic nephropathy. However, in our study the variability of serum lipids did not influence nephropathy outcomes probably due to significantly or insignificantly reduced variability of serum lipids by SGLT2i. SGLT2i treatment reduced ACR to half of baseline (49.2 → 25.6 mg/gCr), and significantly decreased the prevalence of nephropathy.

The reduction of glycemic variability by SGLT2i may have anti-oxidant and anti-inflammatory action and ameliorate endothelial cell alterations (8, 22). We measured hsCRP every three months in two diabetic cohorts for the evaluation of chronic inflammation. The hsCRP at baseline and endpoint in both diabetic cohorts was highly related with BMI (p < 0.001), revealing obesity-induced inflammatory environment for which SGLT2i might be beneficial. The SGLT2i cohort had insignificantly higher hsCRP than patients without SGLTi all the time, and was exposed to active chronic inflammation, because SGLT2i cohort was most obese. However, SGLT2i could not reduce hsCRP significantly. Any parameters of hsCRP did not have significant influence on neuropathy and nephropathy, although mean hsCRP had insignificant negative influence on median nerve amplitude (p = 0.053) and eGFR (p = 0.052). More sensitive markers of chronic inflammation and oxidative stress might be required to detect the benefit of SGLT2i for neuropathy and nephropathy *via* the suppression of chronic inflammation. The somoking and alcohol consumption may influence the neuropathy and nephropathy (29). Because there were no differences between two diabetic cohorts in prevalences (%) of smoking (no smoke/exsmoker/smoker) at the baseline (40/35/25 vs. 37.0/24.7/38.4) and endpoint (40/35/25 vs. 37.0/27.4/35.6) nor alcohol habit at the baseline (17.5 vs. 24.7) and endpoint (15.0 vs.23.3), we could neglect the influence of smoking and alcohol consumption on the present results.

The SGLT2i exerted robust protection against neuropathy and nephropathy over other OHAs. ARB and statin were beneficial for ACR and WPT, respectively. Because prescription rates of both drugs between two diabetic cohorts were similar, the influence of ARB and statin on the benefit of SGLT2i for neuropathy and nephropathy could be neglected.

### Strengths and Limitations

The novelty of the present study is that for the first time the improvement of some NOMs and Z-score of neurophysiological tests by SGLT2i in type 2 diabetes was clarified, and this was the only study that has measured the main risk factors of diabetic microvascular complication, monthly or every 3 months. Therefore, the mean and CV of clinical factors causing neuropathy and nephropathy were representative; the benefits of reduced mean level and variability of glycemia and extraglycemic factors by SGLT2is were reliably evaluated by the multiple regression analysis.

This study has some limitations. Firstly, we used CV of monthly measured HbA1c and CPPG as a glycemic variability; however, there is little consensus regarding the optimal method of assessing glycemic variability. Although CGM provides unique parameters of glycemic variability in type 2 diabetes mellitus (30), in long term follow-up study repeated CGM is impractical. Secondly, due to a lack of a clear definition of long-term glycemic variability, the observation period in studies varied considerably, and most observation periods were a couple of years. A longer observation period may not always be better because of the natural deterioration of patient’s condition (31). Therefore, our observation period of three years appears to be appropriate. There is also differences between the BMI of diabetes groups. The prospective follow-up study of a large number of patients and better matched for weight and BMI may be required to reinforce the present results and establish the neurological and renal protection by SGLT2i.

Thirdly, although the benefit of SGLT2i for neuropathy and nephropathy was exhibited under modest glycemic control, we could not know whether SGLT2i exerted protection against these diabetic complications under poor or good glycemic control.

Therefore, clinical trials should preferably be designed for long periods or include patients with low previous glycaemic exposure to distinguish trial effects from those of the metabolic memory and also include those with poor or moderate glycemic control.

Lastly, because we employed only three out of seven SGLT2is on the market, we could not determine whether the benefit of three SGLT2is is the class effect of SGLT2i or not.

In conclusion, under obesity-induced chronic inflammation SGLT2i ameliorated diabetic neuropathy and nephropathy in modestly controlled patients with type 2 diabetes by reducing glycemic variability and mean level of extraglycemic parameters independent of HbA1c levels.

## Data Availability Statement

The raw data supporting the conclusions of this article will be made available by the authors, without undue reservation.

## Ethics Statement

Written informed consent was obtained from all subjects based on the Declaration of Helsinki. The ethics committee of the Ishibashi Clinic approved the protocol of the present research. The patients/participants provided their written informed consent to participate in this study.Author Contributions

## Author Contributions

FI designed the study, researched data, and wrote the entire manuscript. MT advised on the statistical analysis, interpreted the results, and reviewed and revised the whole manuscript. AK performed all statistical analyses. FI and MT are the guarantors of this work, and, as such, had full access to all data in the study and take responsibility for the integrity of the data and the accuracy of the data analysis and interpretation. All authors contributed to the article and approved the submitted version.

## Conflict of Interest

The authors declare that the research was conducted in the absence of any commercial or financial relationships that could be construed as a potential conflict of interest.

## Publisher’s Note

All claims expressed in this article are solely those of the authors and do not necessarily represent those of their affiliated organizations, or those of the publisher, the editors and the reviewers. Any product that may be evaluated in this article, or claim that may be made by its manufacturer, is not guaranteed or endorsed by the publisher.

## Abbreviations

ACR, albumin-to-creatinine ratio; ARB, angiotensin receptor blocker; BMI, body mass index; CCM, corneal confocal microscopy; CGM, continuous glucose monitoring; CNF, corneal nerve fiber; CPPG, casual postprandial plasma glucose; CPT, cold perception threshold; CV, coefficient of variation; CVR-R, coefficient of variation of R-R interval; DBP, diastolic blood pressure; eGFR estimated glomerular filtration rate; HDL high density lipoprotein; hsCRP, high sensitivity C reactive protein; LDL, low density lipoprotein; MCV, motor nerve conduction velocity; NCV, nerve conduction velocity; NDS, neuropathy disability score; NOM, neuropathy outcome measure; OHA, oral hypoglycemic agent; SBP, systolic blood pressure; SCV, sensory nerve conduction velocity; SGLT2i, sodium-glucose cotransporter-2 inhibitor; VPT, vibration perception threshold; WPT, warm perception threshold.
